# The value of multimodal treatment in anaplastic thyroid cancer patients with distant metastasis

**DOI:** 10.1186/s12893-024-02375-6

**Published:** 2024-03-04

**Authors:** Hongen Guo, Hanqing Lin

**Affiliations:** 1 Department of Dermatology, Dermatology Hospital of Fuzhou, Fuzhou, Fujian Province PR China; 2 Department of Otolaryngology, The First Affiliated Hospital of Fujian Medical University, No. 20 Chazhong Road, Fuzhou, 350005 Fujian Province PR China

**Keywords:** Anaplastic thyroid Cancer, Distant metastasis, Combination treatment, Prognosis

## Abstract

**Background:**

Anaplastic thyroid cancer (ATC) is a rare and aggressive malignancy with a poor prognosis, particularly in patients presenting with distant metastasis (DM). This study aimed to assess the effect of combination treatment strategies on survival in ATC patients with DM.

**Methods:**

A retrospective analysis was conducted using data from the Surveillance, Epidemiology, and End Results (SEER) database to identify primary ATC cases with DM at diagnosis. Univariate and multivariate Cox proportional hazards regression analyses were performed to identify independent risk factors for survival.

**Results:**

Of the 315 ATC patients with DM included in the study, surgery to the primary tumor, radiotherapy, chemotherapy, and lung metastasis were identified as independent risk factors for survival. Patients who received primary tumor surgery plus chemotherapy or surgery plus chemoradiation exhibited a superior outcome compared to those who received only one treatment modality.

**Conclusion:**

Our findings suggest that a combination treatment approach, particularly surgery combined with radiotherapy or surgery combined with chemoradiotherapy, may provide the most optimal treatment option for ATC patients with DM. These results may provide some evidence for clinical decision making, but larger sample cohorts are still needed for validation.

## Introduction

Thyroid cancer is the most prevalent type of endocrine cancer, and its incidence has tripled over the last three decades [1]. Among thyroid cancers, anaplastic thyroid carcinoma (ATC) is the most aggressive cancer type [2], with a median survival of only four months and less than half of patients surviving beyond six months [3]. Distant metastasis (DM) occurs in approximately 50% of patients at the time of diagnosis and is a crucial prognostic factor for disease-specific mortality [4].

Various anti-cancer treatments, including surgery to the primary tumor, radiotherapy, and chemotherapy, have been associated with improved survival in ATC patients [5]. Current guidelines recommend both aggressive therapy and palliative care for ATC patients with DM. However, the effectiveness of combination therapy in patients with DM is still uncertain [6, 7]. This study aimed to evaluate the effectiveness of multimodal treatment strategies for ATC patients with DM using the Surveillance, Epidemiology, and End Results (SEER) program.

## Methods

### Database and case selection

Population-based data for this study were obtained from the SEER 17 database, which covers approximately 26.5% of the US population. Patients diagnosed with metastatic ATC diagnosed in 2010 and 2020 were identified using SEER*Stat software (version 8.4.1; seer.cancer.gov/seerstat). Thyroid cancer was identified using ICD-O-3 codes (C79.3), and ATC was identified using ICD-O-3 histology codes (8021–8035) [8]. Squamous cell carcinoma (8070–8076) was also included as ATC according to the latest WHO classification [9]. The following demographic and clinical variables were collected: age, gender, race, year of diagnosis, TNM stage, distant metastatic sites (bone, brain, liver, lung, distant lymph node), treatment (surgery to primary site, radiotherapy, and chemotherapy), overall survival (OS), cancer-specific survival (CSS), and survival time. Tumor diameter and extension were recorded based on the “Collaborative Stage (CS) tumor size” and “CS extension” for patients diagnosed before 2015, and “Tumor size summary” and “Extent of Disease (EOD) primary tumor” were recorded for patients diagnosed after 2015. The T stage of patients was adjusted to AJCC 8th edition according to tumor size and extension [10]. The surgical approach on the primary site was defined according to “Site-Specific Surgery of Primary Site Codes,” with code 40 (subtotal or near-total thyroidectomy), 50 (total thyroidectomy), and 80 (thyroidectomy, not otherwise specified) defined as thyroidectomy. Code 00 was defined as no surgical treatment on thyroid, and other codes were defined as non-thyroidectomy. According to the SEER manual, ‘beam radiation’ is defined as ‘beam radiation directed to cancer tissue’. Therefore, patients coded as “beam radiation” in this database were considered to have received radiotherapy. Only patients diagnosed with their first malignancy were included, and those without distant metastasis at initial diagnosis, unknown demographic characteristics and treatment data, or unknown involvement of distant sites were excluded.

### Statistical analysis

The primary outcomes of this study were cancer-specific survival (CSS) and overall survival (OS). Kaplan-Meier survival curves and the log-rank test were employed to compare the CSS and OS between groups. Multivariate Cox regression analysis was used to identify independent prognostic factors, with hazard ratios (HRs) calculated along with 95% confidence intervals (CIs).

All statistical tests were two-sided, with *P* values of < 0.05 considered statistically significant unless otherwise stated. Kaplan-Meier analysis was performed using Graphpad Prism 9 (Dotmatics). Multivariate Cox regression analysis was conducted using R version 4.2.1 (R Foundation for Statistical Computing).

## Results

### The basic characteristics of patients

Table 1 presents the baseline clinical characteristics of the 315 patients included in this study. The median age at diagnosis was 70 years, and approximately half of the patients were female (50.79%) and non-Hispanic white (57.78%). The majority of patients were at T4 (73.65%) and N1 (74.92%) stage, with 250 (79.37%) exhibiting extrathyroidal extension. The most common metastatic site was the lung, identified in 266 (84.44%) patients, and 71 (22.54%) patients exhibited multiple organ metastases. Of the 315 patients, 113 (35.87%) underwent a surgical procedure, 183 (58.10%) received radiotherapy, and 149 (47.30%) received chemotherapy.

**Table 1:** Baseline clinical characteristics of 315 ATC patients with DM

	No. patients (%)
Gender	
Female	160 (50.79%)
Male	155 (49.21%)
Age at diagnosis	
Median[min-max]	70.00[23.00,90.00]
Race	
Non-Hispanic White	182 (57.78%)
Hispanic White	53 (16.83%)
Black	20 (6.35%)
Others	60 (19.05%)
T Stage ^1^	
T1/2	17 (5.40%)
T3	66 (20.95%)
T4	232 (73.65%)
N Stage	
N0	79 (25.08%)
N1	236 (74.92%)
Tumor Diameter	
≤ 6 cm	123 (39.05%)
> 6 cm	192 (60.95%)
Tumor Extension	
Limited to Thyroid	65(20.63%)
Extrathyroidal Extension	250 (79.37%)
Distant Lymph Node Metastasis	
No	215(68.25%)
Yes	100(31.75%)
Bone Metastasis	
No	246(78.10%)
Yes	69(21.90%)
Brain Metastasis	
No	296(93.97%)
Yes	19(6.03%)
Liver Metastasis	
No	287(91.11%)
Yes	28(8.89%)
Lung Metastasis	
No	49(15.56%)
Yes	266(84.44%)
Multiple Organ Metastases	
No	244(77.46%)
Yes	71(22.54%)
Marital status	
Single (never married)	40(12.70%)
Married	189(60.00%)
Divorced/Separated/Widowed	83(26.35%)
Unknown	3(0.95%)
Surgery to Primary Site	
No	202 (64.13%)
Non-thyroidectomy	35 (11.11%)
Thyroidectomy	78 (24.76%)
Radiotherapy	
No	132 (41.90%)
Yes	183 (58.10%)
Chemotherapy	
No	166 (52.70%)
Yes	149 (47.30%)

^1^ Refined to AJCC 8th edition according to tumor extension and tumor diameter

### The metastatic pattern and survival of patients

**Fig. 1:**
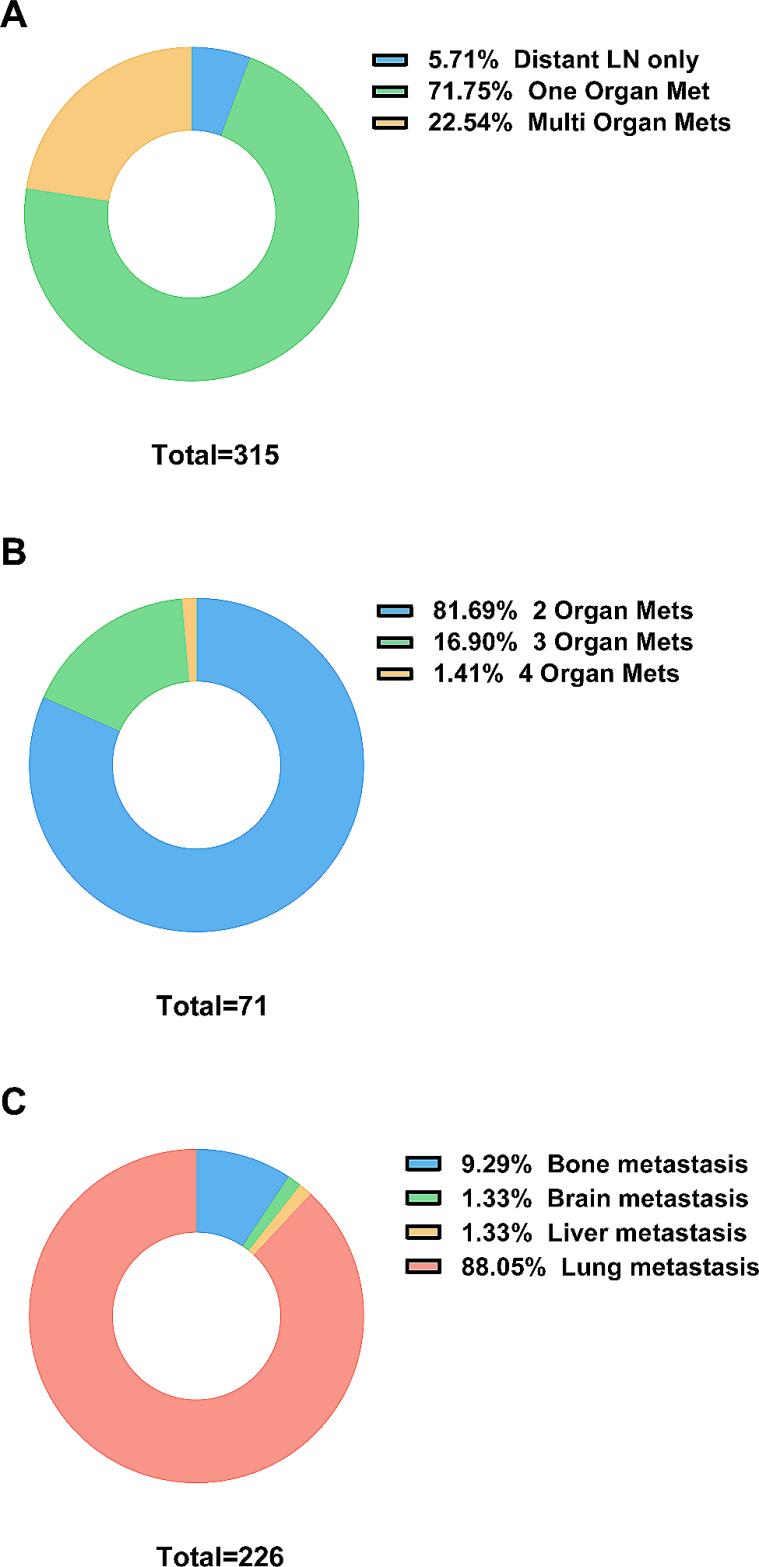
Pie charts showing the metastatic patterns of ATC patients. (**A**) Metastatic pattern of distant lymph node and distant organ in all 315 patients. (**B**) Number of organs involved of 71 patients with multiple organ metastases. (**C**) Metastatic site of 226 patients with one organ metastasis. LN: lymph node. Met/Mets: metastasis/metastases

Among the 315 patients included in this study, 297 had distant organ metastasis, while 18 (5.71%) had only distant lymph node (LN) metastasis (Fig. 1A). Of the 71 patients with multiple organ metastases, 58 (81.69%) had two distant organ metastases, 12 (16.90%) had three distant organ metastases, and only one (1.41%) had four organ metastases (Fig. 1B). Among the 226 patients with single organ metastasis, lung metastasis was the most common, with 199 (88.05%) presenting with lung metastasis, 21 (9.29%) with bone metastasis, and 3 (1.33%) each with brain and liver metastasis (Fig. 1C).

**Fig. 2:**
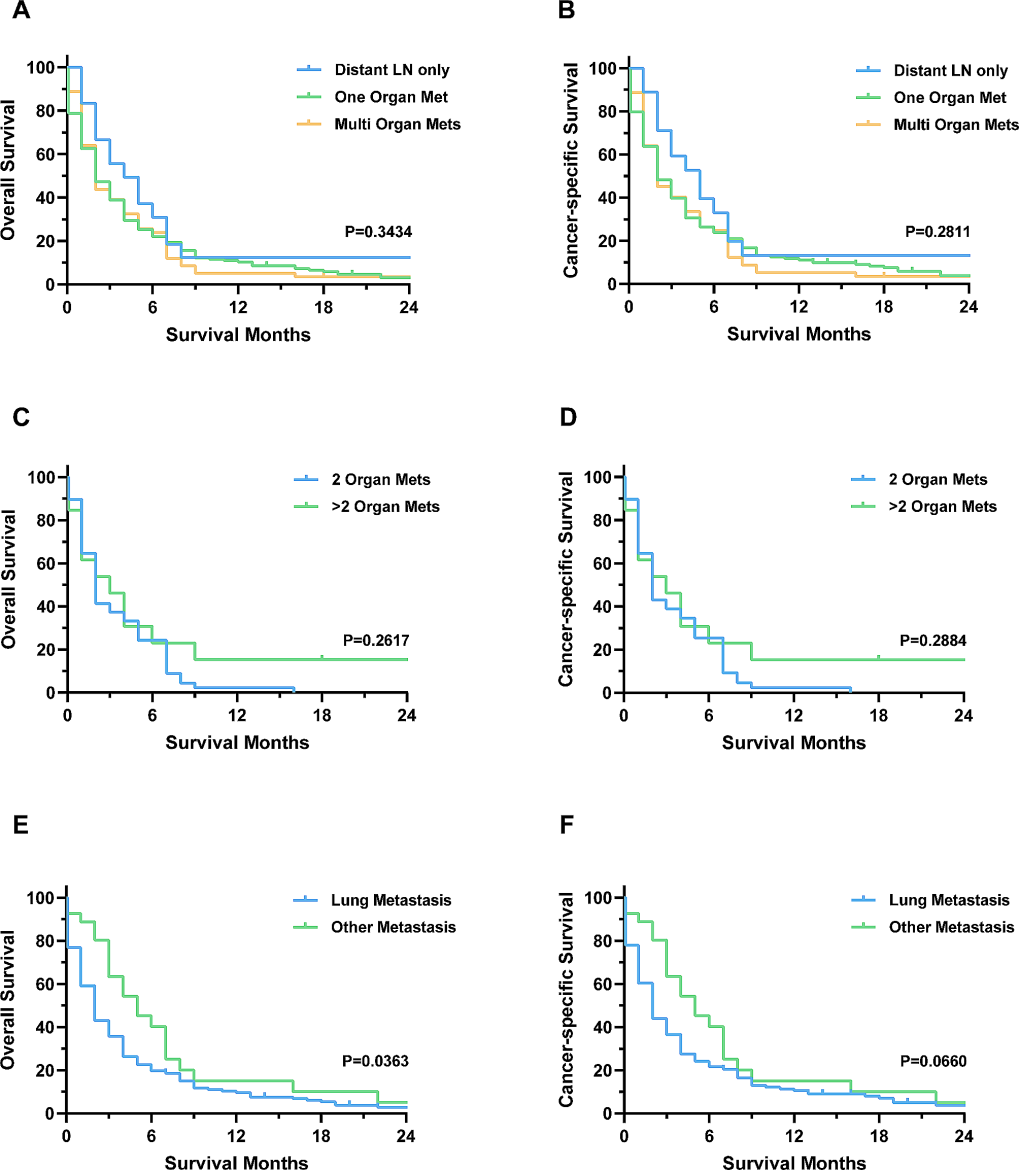
Overall survival (OS) and cancer-specific survival (CSS) of patients with different metastatic patterns. (**A**-**B**): OS and CSS of patients with distant lymph node or organ metastasis. (**C**-**D**): OS and CSS of patients with multiple organ metastases. (**E**-**F**): OS and CSS of patients with single organ metastasis

We subsequently assessed the survival of patients with different metastatic patterns. Our analysis revealed no significant difference in OS and CSS among patients with only distant LN metastasis, single distant organ metastasis, and multiple distant organ metastases (Fig. 2A-B). The number of multi-organ metastases was not significantly associated with patient prognosis (Fig. 2C-D). However, among patients with single distant organ metastasis, OS was worse in those with lung metastasis (Fig. 2E), and this trend was also reflected in CSS (Fig. 2F).

### Analysis of prognostic factors for OS and CSS in ATC patients with DM

We performed univariate Cox regression analysis on all variables to examine the impact of different factors on OS in ATC patients with DM. Our data indicated that being male, younger than 65 years old, having a tumor diameter less than 6 cm, no lung metastasis, undergoing surgery to the primary site, and receiving radiotherapy or chemotherapy were associated with favorable OS. In multivariate Cox regression analysis, all factors except gender were found to be independent prognostic factors affecting OS in ATC patients with DM (Table 2). The factors affecting CSS were similar to those affecting OS, with age, tumor diameter, lung metastasis, surgery, radiotherapy, and chemotherapy identified as independent risk factors (Table 3).

**Table 2:** Univariate and multivariate Cox regression models of factors associated with OS

Characteristics	Univariate			Multivariate		
	HR	95% CI	*P* value	HR	95% CI	*P* value
Gender						
Female	reference			reference		
Male	0.72	0.57–0.91	0.0064	0.86	0.68–1.10	0.228
Age						
≤65	reference			reference		
>65	1.49	1.16–1.91	0.0015	1.30	1.01–1.68	0.045
Race						
Non-Hispanic White	reference					
Hispanic White	1.21	0.89–1.66	0.23			
Black	1.10	0.68–1.80	0.69			
Others	0.98	0.71–1.34	0.89			
T stage						
T1/2	reference					
T3	1.31	0.73–2.36	0.36			
T4	1.41	0.82–2.44	0.21			
N stage						
N0	reference					
N1	0.91	0.69–1.19	0.47			
Tumor Diameter						
≤6 cm	reference			reference		
>6 cm	1.62	1.27–2.07	0.0001	1.19	1.09–1.29	< 0.0001
Tumor Extension						
Limited to Thyroid	reference					
Extrathyroidal Extension	0.96	0.71–1.29	0.8			
Distant Lymph Node Metastasis						
No	reference					
Yes	1.12	0.87–1.44	0.37			
Bone Metastasis						
No	reference					
Yes	0.85	0.64–1.13	0.27			
Brain Metastasis						
No	reference					
Yes	1.06	0.65–1.73	0.82			
Liver Metastasis						
No	reference					
Yes	0.95	0.62–1.45	0.8			
Lung Metastasis						
No	reference			reference		
Yes	1.54	1.11–2.15	0.01	1.55	1.10–2.17	0.011
Multiple Organ Metastases						
No	reference					
Yes	1.10	0.83–1.46	0.5			
Marital status						
Single (never married)	reference					
Married	0.89	0.62–1.28	0.54			
Divorced/Separated/Widowed	1.30	0.87–1.94	0.21			
Unknown	0.88	0.27–2.90	0.83			
Surgery to Primary Site						
No	reference			reference		
Non-thyroidectomy	0.64	0.44–0.94	0.02	0.73	0.50–1.07	0.108
Thyroidectomy	0.51	0.38–0.67	< 0.0001	0.59	0.44–0.79	< 0.0001
Radiotherapy						
No	reference			reference		
Yes	0.60	0.48–0.77	< 0.0001	0.70	0.54–0.91	0.007
Chemotherapy						
No	reference			reference		
Yes	0.39	0.30–0.50	< 0.0001	0.54	0.42–0.70	< 0.0001

**Table 3:** Univariate and multivariate Cox regression models of factors associated with CSS

Characteristics	Univariate			Multivariate		
	HR	95% CI	*P* value	HR	95% CI	*P* value
Gender						
Female	reference			reference		
Male	0.71	0.56–0.90	0.0049	0.85	0.66–1.09	0.183
Age						
≤65	reference			reference		
>65	1.50	1.16–1.93	0.0016	1.31	1.01–1.70	0.046
Race						
Non-Hispanic White	reference					
Hispanic White	1.19	0.86–1.64	0.28			
Black	0.99	0.59–1.67	0.98			
Others	0.96	0.70–1.33	0.81			
T stage						
T1/2	reference					
T3	1.29	0.72–2.33	0.39			
T4	1.36	0.79–2.34	0.27			
N stage						
N0	reference					
N1	0.90	0.68–1.18	0.43			
Tumor Diameter						
≤6 cm	reference			reference		
>6 cm	1.66	1.27–2.14	< 0.0001	1.20	1.10–1.30	< 0.0001
Tumor Extension						
Limited to Thyroid	reference					
Extrathyroidal Extension	0.94	0.70–1.27	0.69			
Distant Lymph Node Metastasis						
No	reference					
Yes	1.13	0.87–1.45	0.36			
Bone Metastasis						
No	reference					
Yes	0.88	0.66–1.17	0.37			
Brain Metastasis						
No	reference					
Yes	1.10	0.67–1.80	0.70			
Liver Metastasis						
No	reference					
Yes	0.94	0.61–1.45	0.77			
Lung Metastasis						
No	reference			reference		
Yes	1.57	1.12–2.21	0.0089	1.58	1.12–2.24	0.009
Multiple Organ Metastases						
No	reference					
Yes	1.13	0.85–1.50	0.39			
Marital status						
Single (never married)	reference					
Married	0.90	0.62–1.31	0.58			
Divorced/Separated/Widowed	1.34	0.89–2.08	0.17			
Unknown	0.93	0.28–3.09	0.91			
Surgery to Primary Site						
No	reference			reference		
Non-thyroidectomy	0.60	0.41–0.90	0.01	0.69	0.46–1.03	0.067
Thyroidectomy	0.52	0.39–0.69	< 0.0001	0.61	0.45–0.81	0.001
Radiotherapy						
No	reference			reference		
Yes	0.59	0.47–0.76	< 0.0001	0.69	0.53–0.90	0.005
Chemotherapy						
No	reference			reference		
Yes	0.39	0.30–0.50	< 0.0001	0.55	0.42–0.71	< 0.0001

### The treatment strategy of ATC patients with DM

**Fig. 3:**
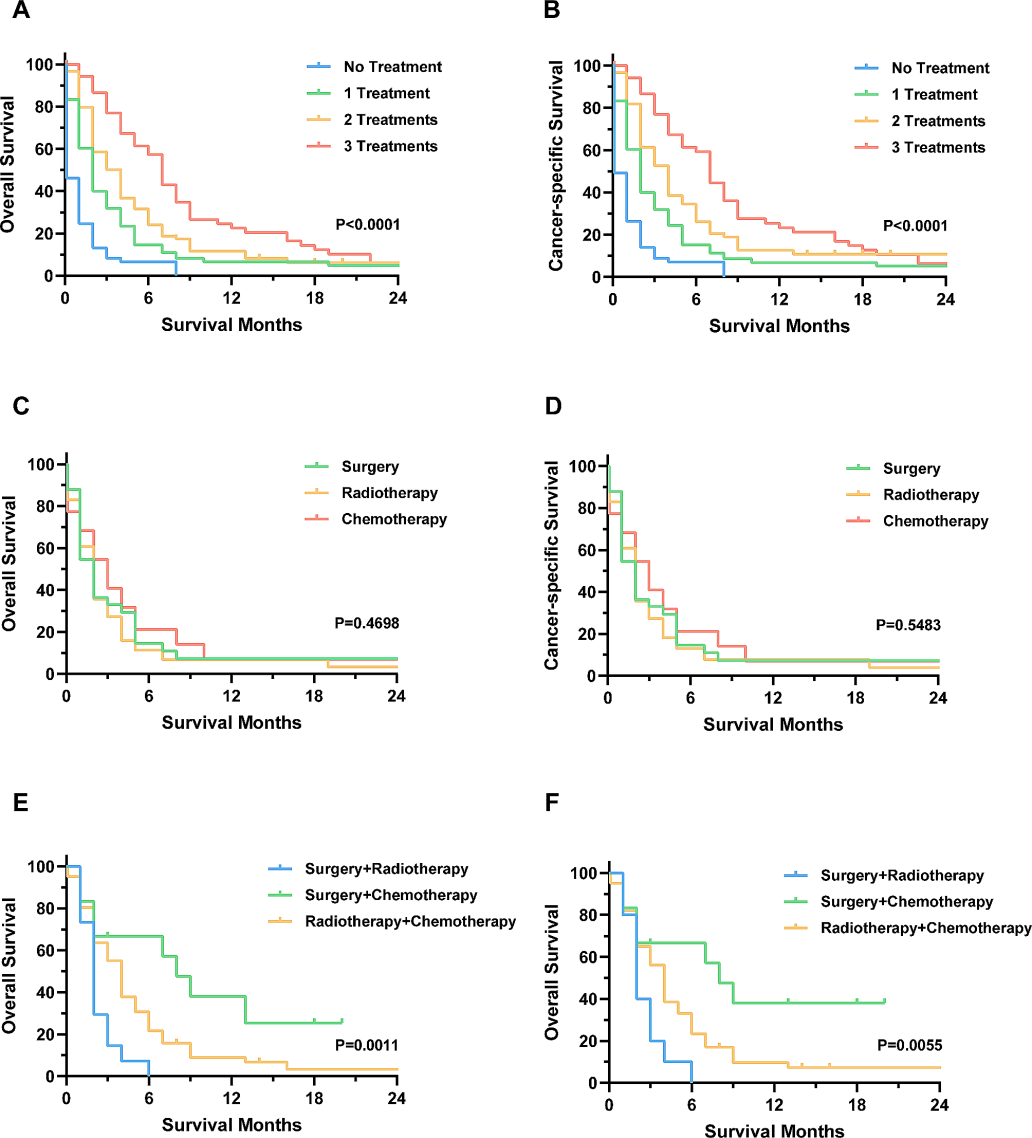
OS and CSS of distant metastatic ATC patients received various treatment strategies. (**A**-**B**): OS and CSS of all patients stratified by the number of treatments received. (**C**-**D**): OS and CSS of patients received single treatment. (**E**-**F**): OS and CSS of patients received multimodal treatments

We further investigated the prognosis of patients receiving different treatment regimens, given that surgery on the primary site, radiotherapy, and chemotherapy were all associated with better survival. Patients who received treatment had better OS and CSS than those who did not receive treatment, and those who received multimodal therapy had better outcomes than those who received single therapy. The median OS and CSS of patients who received no treatment, single treatment, two treatments, and three treatments were both 0.1, 2, 4, and 7 months, respectively (Fig. 3A-B).

We then examined whether there were prognostic differences between the different treatment strategies. Our data showed no differences in patients who received single treatment (Fig. 3C-D). However, among patients who received multimodal therapy, surgery on the primary site plus chemotherapy (median OS and CSS were 8 months) had a prognosis comparable to that of patients who received surgery on the primary site plus chemoradiotherapy and better than those who received surgery on the primary site combined with radiotherapy (median OS and CSS were 2 months) as well as those in the chemoradiotherapy group (median OS and CSS were 4 months) (Fig. 3E-F).

## Discussion

ATC is a rare form of thyroid tumor with an extremely poor prognosis and a high rate of distant metastasis. Previous studies have shown that nearly half of the patients present with DM [8, 11]. Current guidelines recommend surgery, radiotherapy, and chemotherapy for patients with DM from ATC, but it remains unclear whether combination therapy can further improve patient prognosis [6, 7]. In this study, we retrospectively analyzed a large cohort from the SEER database to demonstrate the value of multimodal treatment for the survival of ATC patients with DM.

The impact of metastatic patterns on thyroid cancer prognosis is still controversial. Some studies have reported that the lung is the most common metastatic site for thyroid cancer [12, 13], Matsuzu and colleagues found that patients with lung metastasis had a worse prognosis than those with metastasis to other organs in papillary thyroid carcinoma [14], while Sampson et al. reported that patients with lung metastasis had a better survival than those with bone metastasis [15]. Studies by Vuong and Shao et al. concluded that patients with multiple distant organ metastases had a worse prognosis [8, 16]. However, these studies often include multiple pathological types in the analysis, with varying prognoses. No study has been conducted to evaluate whether metastatic patterns affect the prognosis of patients with ATC. Our study found that lung metastasis was the most common in patients with ATC, with 71 patients (22.54%) having multiple organ metastases and 18 patients (5.71%) having only distant lymph node metastasis. Our analysis showed that patients with lung metastasis had worse OS than those with metastasis to other organs, while other distant metastatic patterns were not significantly associated with survival. This may be due to the small number of patients with different distant metastasis patterns, making it difficult to reach statistically significant differences in subgroup analysis, given the median survival of ATC with DM is only 3 months [17].

Non-treatment factors, such as age, gender, tumor size, and treatment factors, have been reported as predictive factors in ATC patients [5, 18, 19]. Age has been associated with thyroid cancer-specific survival and is included in the AJCC staging system for patients with differentiated thyroid cancer [20]. In our study, being older than 65 years was a negative prognostic factor, which was consistent with the findings of Gui and Wang et al. [19, 21]. The predictive value of tumor diameter was controversial in different studies [5, 21, 22], potentially due to the different cutoff values used in those studies. Our analysis showed that tumor diameter greater than 6 cm was an unfavorable indicator in ATC patients with DM, which confirmed the results of Glaser et al. [5]. Lung metastasis, which was correlated with shorter OS, was confirmed as an independent risk factor. Treatment factors, including surgery to the primary tumor, radiotherapy, and chemotherapy, were all protective factors for OS and CSS, consistent with previous studies [5, 23].

Given that all treatments were shown to be protective factors for patient survival, our study aimed to investigate the impact of different treatment combinations on patient prognosis. Previous studies have suggested that chemoradiotherapy is more effective than radiotherapy alone in patients with ATC [24], a and data by Song et al. has also suggested that surgery-based multimodal therapy improves patients’ prognosis [25], indicating the potential benefit of multimodal treatment compared to monotherapy for patients with ATC. Our results demonstrated that patients who received a combination of two to three treatments had significantly better OS and CSS than those who received only a single treatment, and that the survival of patients who received any treatment was better than that of untreated patients. We then analyzed different treatment combinations and found that chemoradiotherapy was not as effective as other combinations, while surgery plus chemotherapy had comparable survival to patients receiving all three treatments. Surgical treatment is generally not suitable for all patients due to extensive disease, but complete surgical resection is recommended for tumors limited to the thyroid parenchyma [26]. Although our study concluded that the prognosis of patients is not significantly affected by the status of the surgical margins [27], the determination of the extent of surgical resection remains a key concern, considering the widespread invasion of adjacent organs by ATC. Regarding chemotherapy, doxorubicin combined with taxanes and/or platins has been the standard of care for many years [28], and paclitaxel-based regimens are also recommended for ATC patients [29, 30]. Recent clinical trials have demonstrated that for patients with the *BRAF V600E* mutation, dabrafenib plus trametinib significantly improved survival [31]. Despite the fact that new chemotherapeutic agents and regimens continue to be proposed, the response rate in ATC patients is relatively low, and further research is needed on the pharmacological treatment of ATC patients. Radiotherapy could reduce the risk of local recurrence and regional complications [32], but its benefits to patients with DM remains unclear [33]. The acute toxicity induced by radiotherapy may affect its benefits, and future studies should investigate ways to improve the dose and modality of radiotherapy to reduce its toxic effects while retaining local control.

Our study is the largest study to date providing data on the value of multimodal treatment in ATC patients with DM. Despite its clinical significance, there are also limitations to this study. Firstly, the selection biases of retrospective studies cannot be avoided. Secondly, the SEER database only presents data at the time of diagnosis, and the efficacy of different treatments and changes in tumor parameters during follow-up are not available, which limits further evaluation of the effects of different treatment options. In addition, the SEER database is unable to provide specific regimens for radiotherapy or chemotherapy, including the specific areas targeted by radiation and the chemotherapy regimens. So, it was not clear that all of the radiotherapy was directed at primary thyroid tumor, which also compromises data completeness. Therefore, our findings need to be further validated in real-world data.

## Conclusions

Our study included 315 ATC patients with DM, and the analysis showed no significant difference in OS and CSS between the different metastatic patterns. All treatment regimens were identified as independent protective factors for OS and CSS. Specifically, surgery combined with chemotherapy or surgery combined with chemoradiotherapy was associated with better survival compared to monotherapy or other combination treatment modalities. The study was based on a retrospective analysis of the SEER database, and these findings still need to be validated by a larger sample of cohort studies.

## Data Availability

The SEER database analyzed in the current study are available at https://seer.cancer.gov/.

## Abbreviations

ATC: Anaplastic thyroid cancer
CS: Collaborative stage
CSS: Cancer-specific survival
DM: Distant metastasis
EOD: Extent of disease
HR: Hazard ratio
OS: Overall survival
RT: Radiotherapy
SEER: The surveillance, epidemiology, and end results
